# The mechanism of enriched environment repairing the learning and memory impairment in offspring of prenatal stress by regulating the expression of activity-regulated cytoskeletal-associated and insulin-like growth factor-2 in hippocampus

**DOI:** 10.1186/s12199-020-00929-7

**Published:** 2021-01-15

**Authors:** Su-zhen Guan, You-juan Fu, Feng Zhao, Hong-ya Liu, Xiao-hui Chen, Fa-qiu Qi, Zhi-hong Liu, Tzi Bun Ng

**Affiliations:** 1 School of Public Health and Management, Ningxia Medical University, No.1160, Shengli Street, Xingqing District, Yinchuan, Ningxia China; 2 Department of Occupational Health and Environmental Health, College of Public Health, Xinjiang Medical University, Urumqi, 830011 China; 3 Key Laboratory of Environmental Factors and Chronic Disease Control, No.1160, Shengli Street, Xingqing District, Yinchuan, Ningxia China; 4 School of Biomedical Sciences, Faculty of Medicine, The Chinese University of Hong Kong, Shatin, Hong Kong

**Keywords:** Enriched environment, Prenatal stress, Insulin-like growth factor 2 (IGF-2), Activity-regulated cytoskeletal-associated protein (Arc), Learning and memory, Su-zhen Guan and You-juan Fu are coauthors of the paper.

## Abstract

**Background:**

Prenatal stress can cause neurobiological and behavioral defects in offspring; environmental factors play a crucial role in regulating the development of brain and behavioral; this study was designed to test and verify whether an enriched environment can repair learning and memory impairment in offspring rats induced by prenatal stress and to explore its mechanism involving the expression of insulin-like growth factor-2 (IGF-2) and activity-regulated cytoskeletal-associated protein (Arc) in the hippocampus of the offspring.

**Methods:**

Rats were selected to establish a chronic unpredictable mild stress (CUMS) model during pregnancy. Offspring were weaned on 21st day and housed under either standard or an enriched environment. The learning and memory ability were tested using Morris water maze and Y-maze. The expression of IGF-2 and Arc mRNA and protein were respectively measured by using RT-PCR and Western blotting.

**Results:**

There was an elevation in the plasma corticosterone level of rat model of maternal chronic stress during pregnancy. Maternal stress’s offspring exposed to an enriched environment could decrease their plasma corticosterone level and improve their weight. The offspring of maternal stress during pregnancy exhibited abnormalities in Morris water maze and Y-maze, which were improved in an enriched environment. The expression of IGF-2, Arc mRNA, and protein in offspring of maternal stress during pregnancy was boosted and some relationships existed between these parameters after being exposed enriched environment.

**Conclusions:**

The learning and memory impairment in offspring of prenatal stress can be rectified by the enriched environment, the mechanism of which is related to the decreasing plasma corticosterone and increasing hippocampal IGF-2 and Arc of offspring rats following maternal chronic stress during pregnancy.

## Introduction

Pregnancy is regarded as an important event during woman’s life. Women are more likely to be exposed to life stress events during pregnancy, including work pressure, family conflicts, economic distress, etc. It is worth affirming that the change of physiological state during pregnancy has an effect on fetal development in the embryonic stage [1]. Numerous studies have demonstrated that the fetal period plays an important role in the development of cognition, behavior, and cognitive function in offspring [2, 3]; this view was called “developmental programming hypothesis,” which was first highlighted by Dr. David Barker nearly 30 years [4]. Growing epidemiological studies supports that prenatal stress has an adverse effect on the neural development, cognitive development, emotion, temperament, and mental diseases of offspring, such as causing intellectual developmental disorder, low language ability, mental and psychomotor developmental retardation, etc., which can even continue to have harmful effects after birth [5–9].

At present, extensive researches have shown that cortisol (called corticosterone in rodents) released by stress-induced mothers has been identified as stress hormone of prenatal psychological stress [10]. The excessive corticosterone can enter the fetal body through the placental barrier; mainly the maternal corticosterone can reset the hypothalamic pituitary adrenal (HPA) axis. More seriously, the set point of negative feedback regulation mechanism of HPA axis will disturb the development of HPA axis in the vulnerable period of fetus, resulting in the permanent change of HPA axis activity after birth, which is related to the change of behavior and cognition [11, 12]. Prenatal stress elicits lesions of spatial learning and memory ability, for which the candidate brain region is the hippocampus [13]. The hippocampus is enriched in glucocorticoid receptors which play key roles in adjusting the effects of behavioral stress on hippocampal synaptic plasticity and learning and memory function [14]. Adverse events during pregnancy can cause neurobiological and behavioral defects in offspring, especially some of which involve the morphological structure and functional disorders of the hippocampus [15].

Insulin-like growth factor 2 (IGF-2) is the most complex growth factor of the biological function so far, and the concentration is the highest in the hippocampus. Studies confirmed that IGF-2 is mainly expressed in the fetal period, and its concentration in the body fluctuates in different growth stages of the random body, which highly expresses the IGF family in the adult cerebrum [16]. Recently, IGF-2 has been showed to play an important role in consolidation and strengthening of memory [17, 18]. It can help to improve cognitive function of rodents, further promote memory retention, enhance learning and memory ability, and prevent forgetting [19].

The comprehensive mechanism of IGF-2 is not clear, which may be associated with complex process. But interestingly, one of the newly synthesized proteins required for IGF-2-mediated memory enhancement is activity regulated cytoskeletal-associated protein (Arc; also known as Arg 3.1) [20]. Arc is necessary for long-term plasticity, memories formation, maintenance, and needed in the hippocampus and amygdala to consolidate the ability of spatial memory and the cued-fear conditioning [21]. Therefore, we speculated that IGF-2 and Arc worked together in hippocampus impaired learning and memory function of offspring rats which caused by maternal chronic stress during pregnancy.

It is generally known that the structure and function of the central nervous system is influenced by genetic factors and many external conditions. Environmental factors play a crucial role in regulating the development of brain and behavioral. Therefore, if we can choose a kind of environmental stimulation in early life, it may greatly improve ability of learning and memory of offspring which are impaired due to prenatal stress. An enriched environment (EE) is considered as a combination of intricate physical movements and social stimulation [22, 23], which is a large space equipped with the animal experiences exploration and introduction to an array of objects, including appearance, volume, body weight, odor and texture, renders stimulation of the visual, somatosensory, and olfactory systems. It involves that the experimental animals can selectively enter into the animal’s home cage or secondary exploratory which provide physical exercise, exploration, cognition, and social interaction. It has been demonstrated to strengthen long-term potentiation and augment learning and memory performance [24, 25].

The underlying mechanisms involved that enriched environment can repair the learning and memory impairment of offspring caused by prenatal stress, and the mechanism are not yet fully understood. In this report, it was hypothesized that learning and memory defect of offspring rats induced by maternal chronic stress during pregnancy can be rectified by EE, in part as a result of exposure of the fetus to high levels of corticosterone and the expression change of IGF-2, Arc in hippocampus. Therefore, this study was designed to verify the hypothesis that EE can remediate learning and memory impairment of offspring induced by prenatal stress and analyze the mechanisms of it form the expression of IGF-2 and Arc in the hippocampus of the offspring. The participation of this interrelation in the cognitive alterations induced in the offspring by maternal stress is also addressed.

## Materials and methods

### Animals

Twenty female adult Wistar rats weighing (240~270)g and fifteen male Wistar rats weighing (300~350)g were obtained from the Animal Laboratory Center of Xinjiang Medical University (experimental animals certificate number: SCXK (Xin), 2011-000.). They are all sexually mature and were randomly separated into seven cages (5 rats in each cage, female and male apart) after acclimatizing for 1 week. The research project has been approved by the Ethics Committee of Xinjiang Medical University (XJMU#2011015).

### The model of prenatal stress treatments

**Fig. 1:**
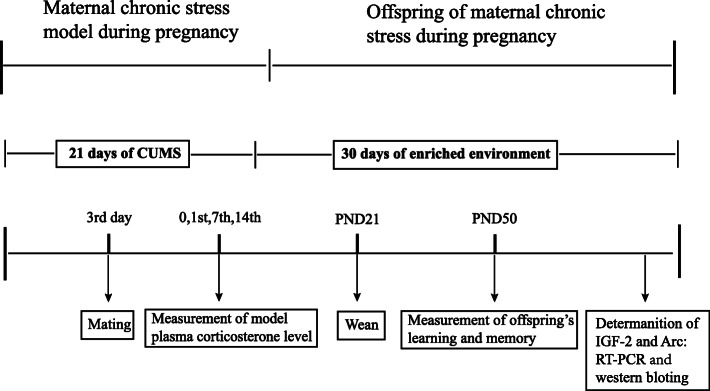
Experimental procedure of learning and memory of the offspring under maternal chronic stress rat during pregnancy

Twenty female rats were randomly divided into two groups (10 rats each group), which are named a prenatal control group (PC group) and a prenatal stress model group (PS group). The prenatal stress rats were performed from the 3rd day after being subjected to chronic unpredictable mild stress (CUMS, the specific procedure was as below 2.3). Before gestation, PS group was mated with a male in one cage, two rats in PC group were mated with a male in a cage. The vaginal walls of all female rats were tested every morning to confirm pregnancy by sperm positivity, designated gestational day 0 (GD 0), then the male and female rats were separated. After gestation, they were forced to return to the original breeding environment. PC rats were housed 5 every cage (1 per cage after GD 18), while PS rats were housed individually (1 per cage). When the rats were mated, every stress factors of CUMS did not suspend everyday. All rats were raised in standard experimental conditions (Fig. 1).

### CUMS procedure

The CUMS procedure was on the basis of a previously described method [26] with minor amendments and supplements. CUMS consisted of exposure to the following stressors in a random order everyday for 21 days: water deprivation for 24 h, food deprivation for 24 h, noise housing (1500 Hz, 92 dB for 1 h), cage tilt (45°, 7 h), forced swimming for 1 h in 31 °C water, squeezing tail for 1 min, behavioral restriction for 4 h, shaking stress (1-s, 30 min), soiled cage (200 ml of water poured into the bedding, 8 h), hot stress in baking oven (42 °C, 5 min). One of ten different stressors was randomly chosen to stimulate every day.

### Measurement of CUMS model through plasma corticosterone concentration

Blood (1 ml) from inner canthus vein were collected from the rats on the first day before stress (baseline) and on the 1st, 7th, and 14th day during stress. Blood samples were centrifuged for 3000 rpm for 20 min at 4 °C, and the obtained plasma was stored at − 35 °C. Corticosterone was measured by using a radioimmunoassay (RIA) kit according to the provided instructions of the manufacturing kit (Coat-A-Count, Diagnostics Products Corporation). The intra-assay variability of the RIA ranged from 3.2 to 4.7%. The plasma corticosterone levels were determined from the measured cortisol value by the following conversion formula: corticosterone concentration = cortisol concentration × 50 [27].

### Dividing the offspring

The day of birth was designated postnatal day 0 (PND 0). Weaning at PND 21, male and female offspring were separated in different cages. Thirty-two offspring of PC group and 32 offspring of PS group were randomly selected. They were assigned to four groups: offspring of PC (OPC, *n* = 16, 8 male vs. 8 female), offspring of PS (OPS, *n* = 16, 8 male vs. 8 female), offspring of PC&EE(OPC&EE, *n* = 16, 8 male vs. 8 female), and offspring of PS&EE (OPS&EE, *n* = 16, 8 male vs. 8 female). All experimental offspring were reared in the same room with free to water and food. The offspring were located in a temperature and humidity controlled laboratory, under a 12/12 day/night cycle (lights on between 07:00 am and 07:00 pm).

### Enriched environmental conditioning

The OPC and OPS offspring without exposure to EE were fed in standard cages (*n* = 4/cage, 60 cm L × 40 cm W × 25 cm H). Offspring with exposure to EE were housed in the large cages (*n* = 8/cage, 90 cm L × 50 cm W × 60 cm H), with one extra level constructed of galvanized wire mesh and connected by ramps of the same material to create two interconnected levels [28]. The EE cages contained wood shavings, a running wheel, a shelter, plastic color toys, and small constructions such as chain, swing, and tunnels. The shelter and running wheel were kept in the cages, particularly the toys and constructions were changed weekly. The feeding boxes and water bottles were moved to different cage points weekly to encourage foraging and explorative behaviors. The four group of offspring were housed under the respective holding different conditions until tested (PND 21–PND 50).

### Measurement of offspring’s learning and memory ability

#### Morris water maze testing

Morris water maze is considered as a model of testing learning and memory function in rats [29]. The water maze apparatus was consisted of a circular pool (1.54 m diameter) made of stainless steel. The pool was filled with water (25 ± 1 °C), which was made opaque by sprinkling the non-sugar milk powder. During Morris water maze training, an escape platform (20 cm diameter) made of stainless steel with a grooved surface was submerged 5 cm under the water level. The training period included 4 trials per day for 3 days. For each trial, the animal was released from 1 of the 4 cardinal compass points (N, S, E, W). The animals were allowed at a maximum of 120 s to locate, and mount the escape platform with a post-trial timeout of 15 s on the platform. In the formal test later, we measured 5 days continuously by recording the time of escape latency and the frequency of locating the platform. The last time of escape latency was the average of the time of escape latency from four different quadrants respectively of the 5 days. After the measurement of escape latency test, the platform was removed, and the offspring were put into the water from the same entry, free swimming was set up for 2 min, and the time while staying in the original platform quadrant through the previous platform was recorded. Another index, the frequency of crossing platform was referred to the number of offspring shuttling the platform through the original position after removing the platform.

#### Y-maze

The Y maze is a device used to measure the learning and memory ability of rats [30]. It consists of three identical arms made of black Plexiglas and multiple extra-maze cues. The three arms were designated Novel, Start, and Other, and they were counterbalanced among rats. On the 1st and 2nd days, after exposing the end of chronic stress, the offspring were tested during training test, and the Novel arm was blocked in order to allow rats to explore the Start and other arms for 15 min. After training, rats were returned to their home chambers (4 h delay) or placed in their home cage outside the testing room (1 min delay). Then rats were returned to the same start location originally determined during training, and allowed them to explore the three arms for 5 min. Rats possess the ability of intact spatial memory, if they showed a preference for the Novel arm because rats have an innate tendency to explore novel environment. All the rats were tested the behavior to measure the number of required training and the rate of correct response in the three arms.

### Tissue collection of offspring

For all subjects, the brain was removed after determination of learning and memory ability. Following anesthesia induced by an intraperitoneal injection of chloral hydrate, the hippocampus was dissected from the brain, placed on a freezing aluminum dissection stage, bisected midway between the septal and temporal poles, then rapidly frozen in liquid nitrogen and frozen on dry ice and stored at − 80 °C. It followed the principle of 50% male and 50% female during the process.

### Quantitative real-time RT-PCR

According to the instructions of RNAsimple Total RNA Kit (TRIzol® Reagent, Life Technologies, USA), total RNA was extracted from 50 to 100 mg hippocampus. Extracted RNA were quantified by nucleic acid protein quantitative instrument. We chose the samples with the A260/280 ratio lies between 1.8~2.0 for further detection. There were two-step reverse-transcription quantitative PCR(RT-qPCR) assay, and both reactions [reverse transcription (RT) and quantitative polymerase chain reaction (qPCR)] were performed in a Chromo 4 Real Time PCR Instrument (MJ Research, USA). cDNA was generated from 600 ng of total RNA in a total volume of 40 μl using a cDNA synthesis kit(ReverttAid First Strand cDNA Synthesis Kit, USA). PCR was performed by using the SYBR® Select Master Mix reagent for performing real-time PCR assays for the genes encoding IGF-2 and Arc. Real-time quantitative PCR (RT-PCR) primers were as follows: IGF-2 (NM_001190162.1): forward, 5′-TACCTCTCAGGCCGTACTTCC-3′, reverse, 5′-TCCAGGTGTCGAATTTGAAGA-3′;Arc(NM_019361.1):forward, 5′-CCCATCTATGAGGGTTACGC-3′, reverse, 5′-TTTAATGTCACGCACGATTTC-3′; β-actin(NM_031144.3): forward, 5′-CCCATCTATGAGGGTTACGC-3′, reverse, 5′-TTTAATGTCACGCACGATTTC-3′. The thermal cycling conditions were as follows: 2 min at 50 °C and 10 min at 95 °C followed by 40 cycles of 95 °C for 15 s and 60 °C for 1 min. The threshold value (Ct) for each sample was set in the exponential phase of PCR, and the ΔΔCt method was used for data analysis. β-action was used as reference gene. The experiment was performed in triplicate.

### Western blotting

After the hippocampal tissue prepared as homogenate, protein concentration was assayed by a bicinchoninic acid (BCA) test (Beijing Tiangen, China). Punches were sonicated in 120–150 μl ice-cold RIPA buffer containing 50 mM Tris-HCl (pH 8.8), 150 mM NaCl, 1% NP-40, and 0.1% SDS (Beijing Applygen Technologies Inc, Beijing, China). Then the homogenate was centrifuged at 12,000×*g* for 5 min, and the supernatant was saved for analysis. Protein concentrations were determined using the BCA assay (Pierce, Rockford, IL). Sample buffer was instantly added to the homogenates, and the samples were boiled for 5 min. Protein extracts (30 μg) were then electrophoresed in 10% SDS-polyacrylamide gels and transferred to polyvinylidene difluoride (PVDF) membranes. Blots were blocked in TBS buffer (50 mM Tris-HCl, pH 7.5, 150 mM NaCl and 0.05% Tween 20) with 5% dry milk and incubated with an anti-Arc/Arg3.1 antibody (1:200; sc-15325, Santa Cruz Biotechnology), anti-IGF-2 antibody (1:200; sc-5622, santa). Blots were incubated with an anti-rabbit secondary antibody conjugated to horseradish peroxidase (1:10,000; 31430, Thermo Scientific) for 1 h. Densitometry was performed based the gray value of the band, and relative protein expression was quantified by densitometry using ChemiScope 3000 (Qing-xiang scientific instrument company, Shanghai, China). To control for inconsistencies in loading, optical densities were normalized to β-actin protein expression. Data for treated animals were normalized to the average value of the naive controls.

### Statistical analysis

The analysis of the experimental date was performed using the Statistical Package for the Social Sciences 13.0, and all graphs were constructed in GraphPad Prism 7.0. All dates were expressed as mean ± SD. Differences between the maternal data and the offspring of four groups in Morris water maze (MWM) performance and plasma corticosterone level of mothers were analyzed using repeated measurement analysis of variance and correlation analysis. One-way ANOVA test was usually used to compare the four groups of data for statistical differences, and the LSD-t test was used to measure the multiple comparison at the two different groups. *P* value less than 0.05 was considered statistically significant.

## Results

### Establishment and confirmation of prenatal stress

**Fig. 2:**
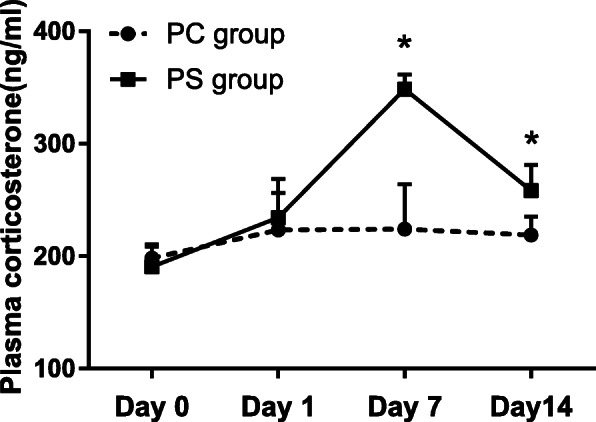
The comparison of plasma corticosterone level between prenatal control group (PC group) and prenatal stress group (PS group). The figure shows that changes in plasma corticosterone level of PS were noted after stress model is established successfully. Each column represents mean ± SD. Number of animals in each group = 10. *P < 0.05 vs. PC group

The repeated measurement analysis of variance revealed that chronic stress had a significant impact on the maternal corticosterone level (*P* = 0.001), and corticosterone level of the PS group obviously changed with stress time (*P* < 0.001). Simultaneously, there was a significant interaction between stress factors and time (*P* = 0.001). LSD-t test showed plasma corticosterone level of the PS group [(348.50 ± 13.24)ng/ml] ascended to the peak value and higher than that of the PC group [(224.00 ± 39.84) ng/ml]during exposing to stress on 7th day (*P* < 0.001). However, the plasma corticosterone of the PS group [(258.38 ± 22.77)ng/ml]declined after exposing the stress on 14th day, but still retained higher than that of the PC group [(218.78 ± 16.68)ng/ml] (*P* < 0.001), illustrating that the PS group stayed in a stressful state. There was no statistical significance of plasma corticosterone levels between the PC group and the PS group at the time of the baseline (*P* = 0.130) and after exposure to stress on the 1st day (*P* = 0.235) (Fig. 2).

### Enriched environment treatment recovered offspring’s plasma corticosterone level induced by prenatal stress

**Fig. 3:**
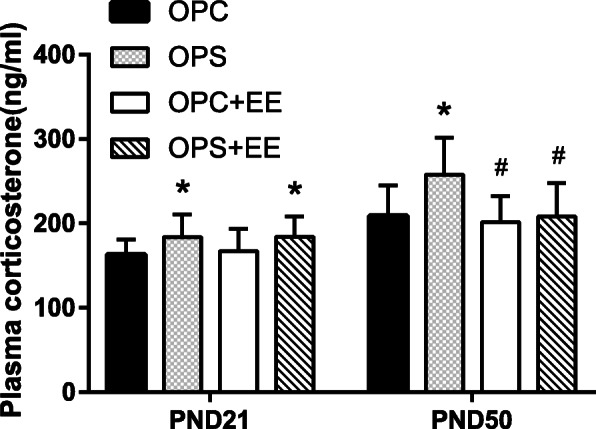
The comparison among OPC, OPS, OPC&EE and OPS&EE in plasma corticosterone level. Differences in corticosterone level were detected among OPC, OPS, OPC&EE, and OPS&EE. The plasma corticosterone level is the highest in OPS group, the lowest in the OPC&EE group. Data were respectively analyzed using One-way ANOVA, followed by LSD-t test to make comparison at the two different groups. Number of animals in each offspring group = 16 (50% male, 50% female)*P < 0.05 vs. OPC. #*P* < 0.05 vs. OPS

High plasma corticosterone level of offspring can frequently be used as an index of physiological response to prenatal stress. In order to examine the offspring’s plasma corticosterone level induced by prenatal stress in the different groups, we found a significant effect on four groups (*P*_(PND 21)_ = 0.048). Specifically, significant exploration of plasma corticosterone level was tested in the OPS [(183.95 ± 26.40)ng/ml] and OPS&EE [(184.17 ± 23.83)ng/ml] groups compared with OPC [(163.15 ± 18.11)ng/ml] (all *P* < 0.05) using LSD-t test. After exposure to the enriched environment, we discovered a significant effect on four groups (*P*_(PND 50)_ = 0.003); LSD-t test stated that plasma corticosterone concentration of the OPS group [(257.88 ± 44.10)ng/ml] was higher than that of the OPC [(208.74 ± 36.40)ng/ml], OPC&EE [(201.61 ± 30.80)ng/ml], and OPS&EE [(208.13 ± 39.83)ng/ml] group (all *P* < 0.05). As shown in Fig. 2, plasma corticosterone level was the highest in OPS group, the lowest in the OPC&EE group, revealing that the OPS group was in a state of stress and it was rectified by exposure to an enriched environment (Fig. 3).

### Enriched environment treatment recovered offspring’s body weight induced by prenatal stress

**Fig. 4:**
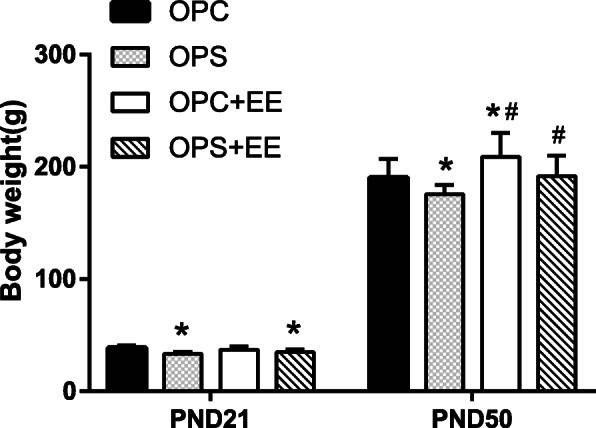
The comparison among OPC, OPS, OPC&EE and OPS&EE in body weight. Differences in body weight were detected among OPC, OPS, OPC&EE, and OPS&EE. The body weight is the lowest in OPS group, the highest in the OPC&EE group; meanwhile, the body weight of OPS&EE is higher than OPS. Data were respectively analyzed using one-way ANOVA, followed by LSD-t test to make comparison at the two different groups. Number of animals in each offspring group = 16 (50% male, 50% female) *P < 0.05 vs. OPC. #*P* < 0.05 vs. OPS

For the sake of investigating whether enriched environment might rectify the growth retardation of offspring, we compared with the body weights of pups on PND 21 and PND 50. One-way ANOVA revealed a significant decrease in OPC [(38.55 ± 2.29)g] and OPC&EE group [(37.07 ± 2.87)g, (*P*_(*PND 21*)_ = 0.025)]. The results showed that exposure to the enriched environment renewed the changes of body weights in OPS [(175.70 ± 8.12)g] and OPS&EE groups [(208.80 ± 21.40)g] compared with OPC [(190.40 ± 16.47)g] (all *P* < 0.05) using LSD-t test. The data showed that body weight was the highest in OPC&EE group and the lowest in the OPS group, indicating that the body weight of OPS and OPC group was increased by exposure to the enriched environment (Fig. 4).

### Exposure to an enriched environment improves offspring’s learning and memory ability in Morris water maze induced by prenatal stress

**Fig. 5:**
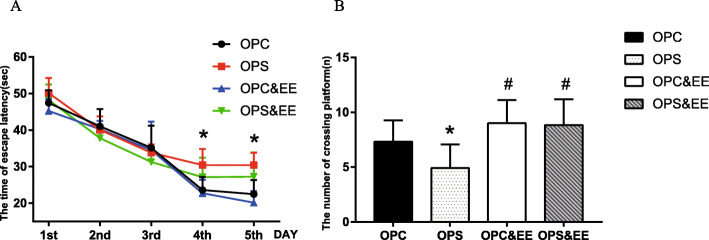
The comparison among OPC, OPS, OPC&EE and OPS&EE in spatial learning and memory in MWM test. Differences in the time of escape latency and the number of crossing platform were detected among OPC, OPS, OPC&EE, and OPS&EE. The time of escape latency was shorten and the number of crossing platform were improved by enriched environment in the OPS. Data were respectively analyzed using repeated measure ANOVA, followed by LSD-t test to make comparison at the two different groups. Number of animals in each offspring group = 16 (50% male, 50% female). (**a** The time of escape latency of the offspring in Morris water maze. **b** The number of crossing platform of the offspring in Morris water maze. *P < 0.05 vs. OPC. #*P* < 0.05 vs. OPS)

The repeated measurement analysis of variance illustrated that prenatal stress had a dramatic impact on the time of escape latency in MWM of offspring (*P* = 0.001), and the time of escape latency significantly changed with different days (*P* < 0.001). At the same time, there was an interaction between time and EE factors (*P* < 0.001). LSD-t test revealed the time of escape latency of PS&EE group was less than PS group (*P* < 0.05) following measurements for 5 days (A), indicating that the OPS group had impaired learning and memory ability, which was enhanced by exposure to an enriched environment. Using one-way ANOVA, we observed significant differences between the four groups at the frequency of crossing platform (*P* < 0.001) (B). The number of crossing platform in PS&EE offspring [(8.83 ± 2.37)] was more than that of PS [(4.92 ± 2.15)] by LSD-t test (*P* < 0.05) (B). The results indicated that environment enrichment increased offspring’ ability of spatial learning and memory ability in MWM test (Fig. 5).

### Exposure to an enriched environment improves offspring’s learning and memory ability in Y-maze induced by maternal chronic stress during pregnancy

**Fig. 6:**
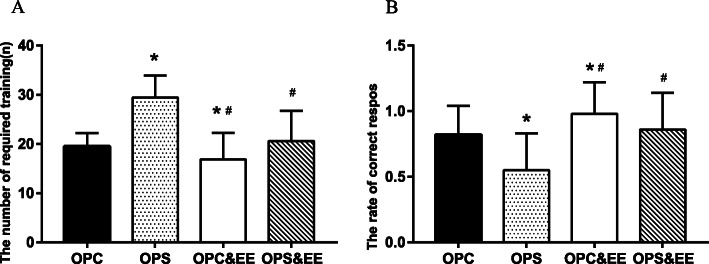
The comparison among OPC, OPS, OPC&EE, and OPS&EE in learning and memory of Y-maze. Differences in the number of required training and the rate of correct response in Y-maze were detected among OPC, OPS, OPC&EE, and OPS&EE. The number of required training and the rate of correct response were improved by enriched environment in the OPS. Data were respectively analyzed using One-way ANOVA, followed by LSD-t test to make comparison at the two different groups. Number of animals in each offspring group = 16 (50% male, 50% female). (**a** The number of required training of the offspring in Y-maze. **b** The rate of correct response of the offspring in Y-maze. *P < 0.05 vs. OPC. #*P* < 0.05 vs. OPS)

One-way ANOVA revealed that exposure to the enriched environment boosted the number of required training and the rate of correct response of Y-maze in the OPS and OPC groups (*P* = 0.000, 0.003). The number of required training (A) in the OPS group [(29.45 ± 4.48)] was more and the rate of correct response (B) [(0.55 ± 0.28)] was less than other groups (all *P* < 0.05). At the same time, the number of required training [(20.60 ± 6.15)] and the rate of correct response [(0.86 ± 0.28)] in Y-maze in OPS group was corrected by exposure to the enriched environment, indicating that environment enrichment increased offspring’s learning and memory ability in Y-maze (Fig. 6).

### Enriched environment treatment improves hippocampal Arc and IGF-2 mRNA expression in offspring induced by prenatal stress

**Fig. 7:**
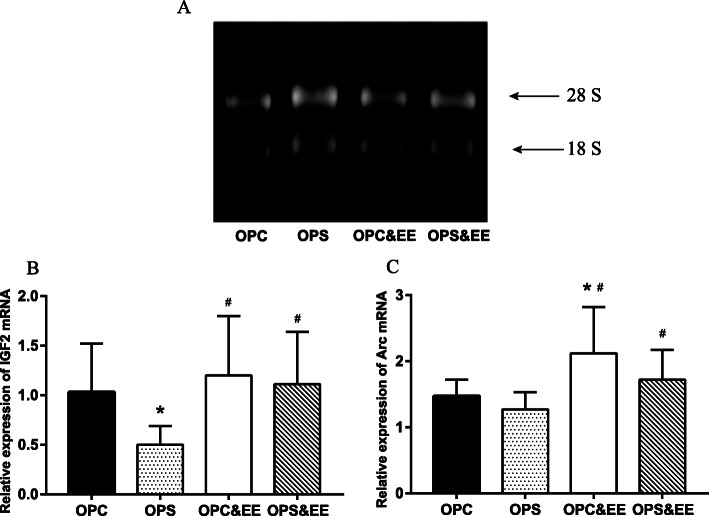
The comparison among OPC, OPS, OPC&EE, and OPS&EE in the IGF-2 mRNA and Arc mRNA expression in the offspring’ hippocampus. The IGF-2 mRNA and Arc mRNA expression of PS offspring were lower than it of PC offspring; however, they were boosted by EE. Data were respectively analyzed using one-way ANOVA, followed by LSD-t test to make comparison at the two different groups. Number of animals in each offspring group = 16 (50% male, 50% female). (**a** The determination of RNA purity. **b** The IGF-2 mRNA relative expression of offspring. **c** The Arc mRNA relative expression of offspring. *P < 0.05 vs. OPC. #*P* < 0.05 vs. OPS)

The extracted RNA was determinated by ultraviolet spectrophotometry, and the results showed that the ratio of A260/A280 was between 1.8~2.0, and there were two clear bands in 28S and 18S by formaldehyde agarose gel electrophoresis. The strip quantity of 28S was twice of 18S, suggested that RNA samples were intact (A). One-way ANOVA revealed that exposure to the enriched environment boosted the average expression of IGF-2 mRNA (B) and Arc mRNA (C) in the OPS and OPC groups (*P* = 0.012, 0.026) through RT-PCR (Fig. 7).

### Enriched environment treatment improves hippocampal IGF-2 and Arc protein expression in offspring induced by prenatal stress

**Fig. 8:**
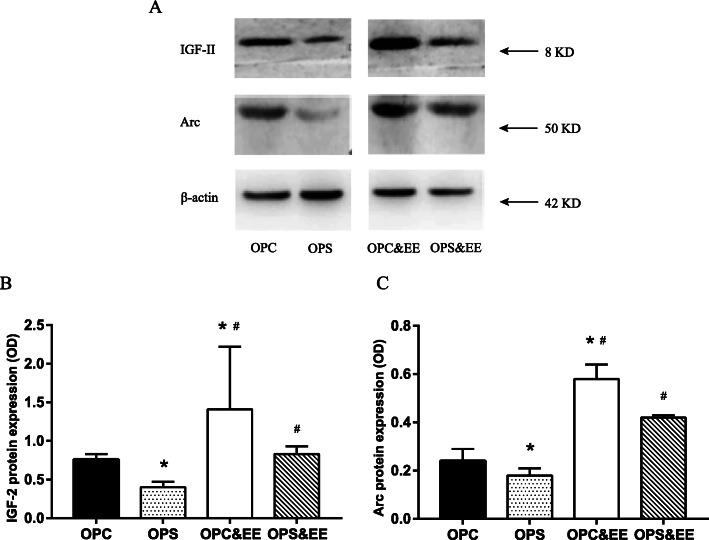
The effects of enriched environment on the hippocampal IGF-2 and Arc protein expression in the offspring due to maternal stress of pregnancy. Differences in IGF-2 and Arc protein expression in the hippocampus were detected among four groups. The IGF-2 and Arc protein expression of PS offspring were lower than that of PC offspring; however, they were increased by EE. Data were respectively analyzed using One-way ANOVA, followed by LSD-t test to make comparison at the two different groups. Number of samples in each offspring group = 4 (50% male, 50% female) (**a** Electrophoresis results of IGF-2 and Arc protein expression in offspring. **b** IGF-2 protein expression in the hippocampus of offspring. **c** Arc protein expression in the hippocampus of offspring. *P < 0.05 vs. OPC. #*P* < 0.05 vs. OPS)

The molecular weight of IGF-2 and Arc were respective 8 kD and 50 kD on the basis of Western blotting (A). Within the average optical density value (OD) displayed, one-way ANOVA revealed a significant difference of hippocampus in IGF-2 (B) and Arc (C) protein among OPC (0.76 ± 0.07, 0.24 ± 0.05), OPS (0.40 ± 0.07, 0.18 ± 0.03), OPC&EE (1.41 ± 0.81, 0.58 ± 0.06), and OPS&EE (0.83 ± 0.10, 0.42 ± 0.01) groups (all *P* < 0.001). Compared with OPS group, the IGF-2 and Arc protein of hippocampus increased after exposure to the enriched environment (Fig. 8).

## Discussion

As we all know, women are more likely to be exposed to life stress events during pregnancy, and these life stress events will lead to a variety of negative emotions such as depression and anxiety during pregnancy and postpartum. Such emotions are not only harmful to the physical and mental health of pregnant women, but also cause changes in the intrauterine environment, and even affect the later growth and development. Due to the inherent difficulties and ethical issues in the research of human beings, the influence of prenatal stress has been widely studied in animal models, especially in rats. Indeed, different types of stressful procedures applied to pregnant rodents are well documented and have been shown to produce a large number of biological and behavioral dysfunctions [31] and pups [9, 32]. In our study, we chose chronic unpredictable mild stress (CUMS) model, initially created by Paul Willner and colleagues [26]; this is a widely used rodent model of depression produced by stress which entail repeated exposure to a series of varying and unpredictable, mild stressors over a continuous period of time, and that usually range from 10 days to 8 weeks. We started mating experiment during 3rd day in 2 days model period. The experimental results showed that the level of plasma corticosterone (stress hormone) in PS group was higher than that in PC group, indicating that the model was established successfully. It has been shown that the increased maternal corticosterone levels and the excessive of this hormone can by-pass the placental barrier, which could increase fetal exposure to maternal corticosterone. Fetal brain overexposure to maternal corticosterone can induce various negative consequences, such as prenatal stress can reduces intrauterine growth and birth weight [33, 34]. In our study, the decrease of body weight of the offspring in PS group may be related to the increase of plasma corticosterone level of mothers. The structure of the central nervous system is very complex, which is determined by genetic factors and various external conditions after birth, and early life is a critical period of neuronal growth, differentiation, and synapse formation. After birth, the newborn remains sensitive to the changes of the environment. Fortunately, a number of events can act positively during the neuro-development of pups, for example, enriched environments. Enriched environment (EE) is that a group of several animals (8–12) live together in a large cage; there are various types of toys and changed frequently. It shows EE consists of enhanced social interactions and offers considerably more opportunities for interactions with non-social stimuli. In contrast, standard environment consists of standard laboratory cages in which 4 to 5 animals are housed together. Our study showed that high plasma corticosterone level of offspring induced by prenatal stress was rectified by exposure to an enriched environment. We also found that birth weight of OPS group was lower than that of OPC group, but it also was boosted by exposure to an EE.

Numerous animal studies have put forward that early life environment have a significant impact on the process of hippocampus-mediated learning and memory ability of offspring. Prenatal stress can alter the synaptic plasticity responsively in hippocampal CA1 region and deteriorate the effects of acute stress on synaptic in offspring [35] and is related with the cognitive impairments in spatial learning and memory ability of offspring [36]. EE was identified to repair abnormal behaviors, for instance, emotional reactivity and spatial learning ability [37–39]. Our study also drew a conclusion that learning and memory ability was closely related to the time of escape latency and the number of crossing the platform in Morris water maze; the frequency of required training and the rate of correct response in Y-maze was redressed after living in an enriched environment. The reason is that EE probably decreased glucocorticoid receptors in hippocampus of offspring after exposure to an enriched environment. As far as we know, the mechanism by which the enriched environment corrects the effects of maternal chronic stress on synaptic plasticity in the hippocampus of offspring of prenatal stress is still unclear. However, the video recording of the Morris water maze and Y-maze experiment was not collected in this study due to the limited experimental conditions at the time. In the future relevant research, we will try our best to keep the video information, which would more vividly confirm the impairment of learning and memory ability in offspring during prenatal stress and exposure to EE can repair this impairment.

The hippocampus is a good source of glucocorticoid receptors, which play an important role in regulating the effects of behavioral stress on learning and memory along with on synaptic plasticity [40]. Furthermore, prenatal stress can alter in early postnatal development of the hippocampal functions and structures that may implicate the mechanisms of cognitive deficits in children. Many studies show that chronic early-life stressors are associated with the structure and function impairments in hippocampus of adulthood [41]. Hippocampal synaptic plasticity, particularly the long-term potentiation (LTP) and long-term depression (LTD), is believed to be the underlying mechanism of learning and memory functions. Other studies also found that prenatal stress elevated the maternal concentration of stress hormones, which negatively affects fetal hippocampal development and definitively prolongs corticosterone response in the offspring when under stress and accounts for the reduced number of corticosteroid receptors in the hippocampus [42]. Thus, prenatal stress can enhance the effect of acute stress on the synaptic plasticity of hippocampus by changing the synaptic plasticity of hippocampus, which may be one of the mechanisms of impairment of spatial learning and memory in offspring.

To determine the mechanisms underlying the effects of maternal stress during pregnancy on cognition in offspring and the intervention effect of EE, we focused on two molecules involved in the memory processes—IGF-2 and Arc. Studying revealed that the IGF-2 and Arc in hippocampus increased after exposure to the enriched environment, compared with OPS group. IGFs, including IGF-2, have been shown that they can cross the blood–brain barrier in some species and also is a polypeptide belonging to the insulin system, which play an important role in normal somatic growth and development, tissue repair, and regeneration. The growth of IGF-2 expression in the hippocampus is needed for the rat inhibitory avoidance memory formation and mouse extinction learning [43], and a bilateral hippocampal injection of recombinant IGF-2 strengthens fear memory retentivity and prevents from forgetting. IGF-2 is endogenously upregulated following learning as a C/EBP target gene and needed in the hippocampus for memory consolidation process [44] and may be particularly suitable as a reinforcer of hippocampal or cortica-dependent memories. Arc is a particularly interesting and promising candidate, which likely underlies the consolidation and reconsolidation of some memory forms. Above all, it can be rapidly transported to activated synaptic zone, where its protein product associates with the dendritic cytoskeletal proteins [45].The Arc protein exists postsynaptic clearance, which works together with the N-methyl-d-aspartic acid receptor complexes (NMDARs). Apart from the HPA axis, signaling through NMDARs is critically involved in learning, memory, and synaptic plasticity [46]. Secondly, while Arc typically shows relatively low expression, its expression in hippocampus is promptly and robustly heightened following with a large number of behavioral performances, including trace and contextual fear condition [47] exploration of a novel environment and spatial learning [48]. Therefore, Arc is one of rapidly regulated translation, usually known to occur at activating synapses and critical for long-term plasticity and memory [49]. Combined with our finding that enriched environment repairing the learning and memory impairment in offspring of prenatal stress correlated with IGF-2 and Arc protein expression, these findings raise the possibility that IGF-2 and Arc can promote synapse-specific translation during LTP production or memory formation [50].

## Conclusions and implications

Taken together, we draw a conclusion that the results revealed in this study provide a strong evidence for extreme sensitivity of enriched environment treatment repaired learning and memory deficits induced by maternal prenatal stress. It can be postulated that a better understanding of IGF-2 and Arc in hippocampus had played an important role in linking the effects of offspring EE with the brain plasticity and the learning and memory dysfunction exposure to prenatal stress. Another factor that is worth considering is that EE also can decrease corticosterone level of offspring resulting from prenatal stress. In view of these findings, the exact mechanism involved remains to be further elucidated.

## Data Availability

The corresponding author can provide the datasets used and analyzed during the current study.

## Abbreviations

IGF-2: Insulin-like growth factor-2
Arc: Activity-regulated cytoskeletal-associated
EE: Enriched environment
CUMS: Chronic unpredictable mild stress
RT-PCR: Real-time quantitative PCR
PS: Prenatal stress
PC group: Prenatal control group
PS group: Prenatal stress model group
GD: Gestational day
PND: Postnatal day
OPC: Offspring of prenatal control group
OPS: Offspring of prenatal stress model group
MWM: Morris water maze
BCA: Bicinchoninic acid
LTP: Long-term potentiation
LTD: Long-term depression
IA: Inhibitory avoidance
